# Mobile health apps for skin cancer triage in the general population: a qualitative study on healthcare providers’ perspectives

**DOI:** 10.1186/s12885-025-14244-3

**Published:** 2025-05-09

**Authors:** Tobias E. Sangers, Marlies Wakkee, Folkert Moolenburgh, Tamar Nijsten, Marjolein Lugtenberg

**Affiliations:** 1 Department of Dermatology, Erasmus MC Cancer Institute, University Medical Center Rotterdam, Rotterdam, 3015 GD The Netherlands; 2 Department of Dermatology, Leiden University Medical Center, Leiden, The Netherlands; 3 Department Tranzo, Tilburg School of Social and Behavioral Sciences, Tilburg University, Tilburg, The Netherlands

**Keywords:** Artificial intelligence, Smartphone apps, Dermatology, Skin cancer

## Abstract

**Background:**

Mobile health (mHealth) applications (apps) integrated with artificial intelligence for skin cancer triage are increasingly available to the general public. Nevertheless, their actual uptake is limited. Although endorsement by healthcare providers (HCPs) is one of the perceived facilitators for using this technology, the perceptions of key HCPs in skin cancer triage towards those apps have not been studied.

**Objectives:**

To explore key HCPs’ perceived risks, benefits, and preconditions for endorsement of mHealth apps for skin cancer triage in the general population.

**Methods:**

An in-depth qualitative online focus group (FG) study was conducted consisting of six focus groups: three with dermatologists and three with general practitioners (GPs). Dutch dermatologists and GPs were selected using purposive sampling based on age, knowledge and previous experience with AI. A total of sixteen dermatologists and seventeen GPs attended 90-minute FGs. Data were analyzed by a multidisciplinary team in a thorough thematic content analysis using multiple phases of coding derived from Grounded Theory.

**Results:**

A total of four main risks, three main benefits, and four main preconditions for endorsement were identified. Risks perceived by HCPs concerned *incorrect diagnoses*, *exclusion of subpopulations*, and *loss of GP autonomy in clinical decision making* and *diagnostic experience*. Perceived benefits were *increased skin cancer awareness*, *facilitation of the early detection of skin cancer*, and a *streamlined patient journey*. Preconditions for endorsement were *evidence-based verification of accuracy*, *integration in clinical practice*, *clarity about liability in case of adverse events*, and *accessible and inclusive app design*.

**Conclusions:**

Although HCPs perceive pivotal risks related to the implementation of mHealth apps, they also foresee important benefits when implemented successfully. In order for HCPs to endorse those apps, emphasis must be placed on integrating accurate mHealth apps with accessible and inclusive design and functionality into clinical practice, factors that currently appear to be largely unmet.

**Supplementary Information:**

The online version contains supplementary material available at 10.1186/s12885-025-14244-3.

## Introduction

Mobile health (mHealth) apps integrated with AI for skin cancer triage have gained increased attention because of their potential to provide patients with accurate lesion assessments and reduce unnecessary referrals [1]. Health insurers in Europe, Australia, and New Zealand are already embracing these apps through reimbursement policies [2–6]. However, public adoption remains limited. Barriers, such as doubts about value and trustworthiness may limit their adoption, while factors like perceived value, developer transparency, and endorsement from healthcare providers may facilitate their usage [7]. Amidst the growing interest in mHealth apps, concerns echo through the scientific and clinical community about their use [8, 9]. Despite the pivotal role of healthcare providers (HCPs), including dermatologists, in facilitating mHealth app adoption, their perspectives on the use of these tools by the general population have received limited attention. This study aims to fill this gap by exploring Dutch key HCPs’ perspectives on the risks, benefits, and conditions for endorsing mHealth apps for skin cancer triage among the public. The study results of this study can inform an improved implementation strategy in healthcare, aligning with the views of both HCPs and the general population.

## Methods

### Setting

The study was conducted within the healthcare system of the Netherlands, where General Practitioners (GPs) play a pivotal role in coordinating and regulating access to specialized care. This stands in contrast to countries where access to specialized care often involves direct patient referrals to specialists without the intermediary role of GPs. Nonetheless, it is important to emphasize that there are significant similarities in how GPs across different healthcare systems approach patients with skin cancer, highlighting shared practices and areas of convergence.

### Study design

We performed a qualitative focus group study among key HCPs in skin cancer triage in the Netherlands, i.e. dermatologists and GPs, to generate a comprehensive overview of their perceptions towards the use mHealth apps for skin cancer triage in the general population [10, 11]. Focus groups were separately organized for dermatologists and GPs and were hosted online using Microsoft Teams due to COVID-19 social distancing measures. This study was designed and reported in accordance with the Standards for Reporting Qualitative Research [12].

### Selection of participants

Using purposive sampling Dutch dermatologists and GPs with varying prior knowledge and/or experience with AI, gender, and age were recruited to participate in the study [10]. Potential participants were informed about the possibility to participate via social media (WhatsApp/Telegram group dermatologist and GP group chats, LinkedIn, Facebook), via e-mail, and via the Dutch Society of Dermatology and Venereology’s newsletter. Participants were offered a €30 gift card for compensation and could apply to participate using a web form.

### Data collection

A topic guide with key questions and prompts was developed to facilitate discussion [10]. This guide drew from prior literature on mHealth adoption in the general population and our research team’s experience in AI and skin cancer triage qualitative research [7, 10, 13]. Participants provided informed consent and completed a brief demographic questionnaire before the 90-minute focus group sessions, led by two medical doctors (MDs), one with expertise in qualitative research (TS, FM).

### Data analysis

We conducted a thorough thematic content analysis within a constructivist paradigm, drawing on elements from Grounded Theory [14, 15]. All focus groups were recorded and transcribed post-consent. Data analysis employed NVivo software (QRS International Pty Ltd, Doncaster, Vic, Australia). Two researchers (TS, FM) independently performed open coding, involving a line-by-line analysis of fractured FG transcripts into unstructured codes [14]. This was followed by axial coding, grouping codes into more abstract ones, yielding initial concepts [14]. After analyzing four focus groups, our multidisciplinary team (TS, FM, ML) determined data saturation had not been reached, after which two additional FGs with dermatologists and GPs were held [16]. After analyzing the additional transcripts the resulting themes and subthemes were discussed and finalized, confirming thematic content saturation within the multidisciplinary team (TS, FM, MW, ML). We employed the constant comparison technique throughout all coding phases to compare existing concepts and themes with new data [14]. Participant characteristics were analyzed using SPSS Statistics v.15 (IBM Corp., Armonk, NY).

## Results

### Characteristics of participants

Group level characteristics of participants are presented in Table 1. Individual participants’ characteristics can be found in supplementary eTable 1.

**Table 1:** Participants’ characteristics. GPs, general practitioners; IQR, interquartile range

	Participants, *n*	Median age (years), (IQR)	Female, *n* (%)	Familiar with AI for skin cancer triage, *n* (%)	Sees AI as an addition to skin cancer care, *n* (%)
Focus group 1 (GPs)	4	32 (32–34)	2 (50%)	0 (0.0%)	3 (75%)
Focus group 2 (GPs)	5	42 (35–53)	2 (40%)	3 (60%)	5 (100%)
Focus group 3 (Dermatologists)	4	49 (42–57)	2 (50%)	4 (100%)	4 (100%)
Focus group 4 (Dermatologists)	5	41 (35–52)	2 (40%)	4 (80%)	4 (80%)
Focus group 5 (GPs)	8	34 (32–35)	6 (75%)	3 (38%)	8 (100%)
Focus group 6 (Dermatologists)	7	39 (33–43)	5 (71%)	6 (86%)	7 (100%)
Total	33	36 (33–42)	19 (58%)	20 (61%)	31 (94%)

**Fig. 1:**
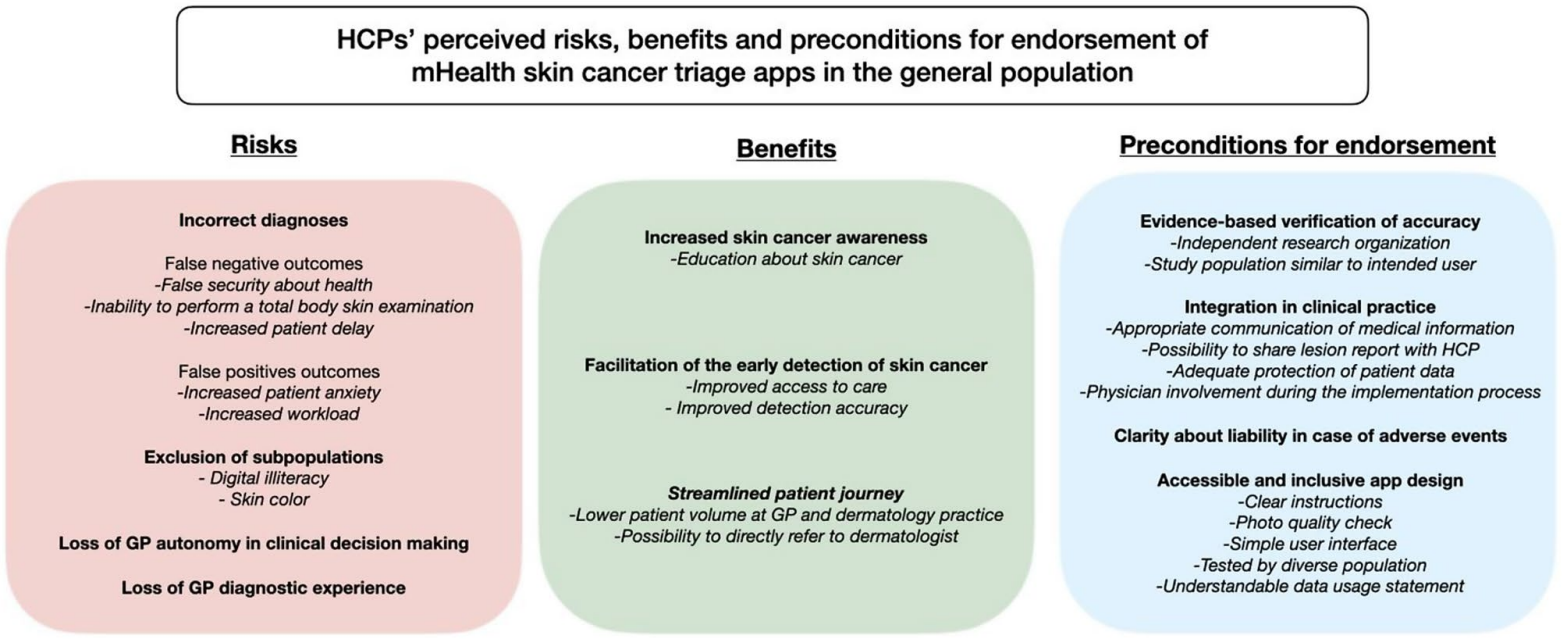
Perceived risks, benefits and preconditions for endorsement of mHealth apps for skin cancer triage used in the general population, as perceived by HCPs

*Views of HCPs towards using mHealth apps for skin cancer triage in the general population.* In total, four main risks and three main benefits towards the use of these apps among the general population and four main preconditions for endorsement were identified (Fig. 1).

### Risks

The first perceived risk towards the use of mHealth apps for skin cancer triage in the general population, were *incorrect diagnoses*. Incorrect diagnoses were considered to lead to false negative, i.e. missed premalignant and malignant lesions by being diagnosed as benign, and false positive, i.e. benign lesions unjustified being diagnosed as premalignant or malignant, outcomes. Regarding false negative outcomes, GPs noted that users might be falsely reassured about a premalignant or malignant skin lesion by an app, leading to *false security about users’ health*. This was not only related to the accuracy of the app but also due to the perceived *inability to perform a total body skin examination with an app.* According to dermatologists and GPs people in the general population are incapable of accurately deciding which lesions are suspicious for skin cancer, thereby increasing the risk of missed diagnoses when used as a tool for skin cancer triage. In line with this concern, dermatologists noted that false negative outcomes might lead to *an increased patient delay* in visiting a doctor, potentially causing skin cancers progression.

*This is, besides the known problems, for me the biggest problem. That not the entire skin is examined. – Derm, FG 8-12.*

Specifically related to false positive outcomes, HCPs mentioned *increased patient anxiety* due to a low specificity of those apps to recognize benign lesions. Additionally, they reasoned that this would lead to patients being referred into care more than necessary, leading to *an increased workload*, as a proportion of patients will be advised to visit a doctor for a skin lesion while there is no need for it.

*And if we want to implement an app on a much larger scale, it’s going to be a lot more [patients referred by an app]. And if we have to go see all those people, I think you’re going to get a lot more care and not necessarily better care. – Derm, FG 12-1*.

A second important perceived risk by GPs was the potential prospect of *excluding specific subpopulations*. This exclusion was, first of all, focused on the *digital literacy* of potential users. Participants explained that people in the general population and patients with low digital literacy might not be able to use mHealth apps, thereby excluding them from any benefits that may be associated with its use. In addition, GPs noted that potential users might be excluded based on *skin colour*. GPs noted that AI algorithms are known to perform better on white skin compared to colored skin.

*Yeah, and I think what you’re going to get more and more in the future is inequity in access to care. – GP FG 24-11.*

*Loss of GPs autonomy in clinical decision making* was identified as the third perceived risk, mentioned by HCPs. They were concerned that patients receiving worrying advise from an app regarding a benign skin lesion may subsequently refuse to be reassured after a physical examination by the GP or other primary healthcare providers and demand a referral to a specialist.

*I can imagine that as a GP it puts you in a tough position, that someone comes in with an app, that they have skin cancer and need to be referred, and then, yes, you are supposed to make a referral. And while you might think very differently about it, or you don’t think it’s suspicious at all, or you see something else, or you want to remove something yourself or do diagnostics in primary care, biopsy or something. That this also limits you. And that you just have to write a referral, because that’s what the patient wants. – GP, FG 23-11.*

The fourth risk, specifically identified for GPs, was *the risk of loss of GP diagnostic experience* regarding skin lesions. When mHealth apps will be implemented as triage before a GP visit, this will lead to significantly fewer patients visiting GPs. As a result, the diagnostic experience of GPs regarding skin lesions was thought to potentially deteriorate over time.

*I’ve already seen a few patients who had been referred, and the referral letter stated that the app indicated an increased risk, so the patient was referred. I didn’t really get the impression that the GP had a personal opinion about it. – Derm, FG 17-12.*

### Benefits

*An increased skin cancer awareness* was identified as the first benefit of mHealth apps for skin cancer triage used in the general population perceived by HCPs.

*Perhaps an indirect good effect of this is that it creates more awareness. So that people will pay more attention to these kinds of things. (…) So that people are more aware of suspicious skin lesions* – *Derm*,* FG 12-1*.

mHealth apps were thought to raise awareness by *educating users about skin cancer* and its risks, e.g. by informing them about the need to perform self-examinations of the skin and by providing information about the multiform appearance of different types of skin cancer. The second identified benefit of mHealth apps for skin cancer triage in the general population among HCPs was *facilitation of the early detection of skin cancer*, supported by two sub-benefits. First, mHealth apps were mentioned to provide users with the possibility to screen a skin lesion for skin cancer at their own discretion, without being limited to the opening hours of the dermatologist or GP practice. HCPs thought that this would lower the barrier to assessing a potentially cancerous lesion, thereby *improving the access to care* and allowing skin cancer patients to be diagnosed at an earlier stage. Second, HCPs considered mHealth apps to potentially *improve skin cancer detection accuracy* in the general population. This improved detection accuracy was thought to empower individual capabilities in skin self-examination and thereby facilitate the early detection of skin cancer.

*I can imagine it’s quite a useful app. It’s probably set very defensively*,* so it might create more work for us (…) but that’s fine — we’ll see people with possible skin cancer sooner. – GP FG 7 − 1*.

*A streamlined patient journey* was identified as the third and final benefit for HCPs, supported by two sub-benefits. They believed that the use of mHealth apps as a triage tool before deciding to visit a doctor could lead to *a lower patient volume*. However, sufficient triage accuracy was seen as an important perceived precondition for this sub-facilitator to become reality. A second potential sub-benefit was the possibility *of referring highly suspicious skin lesions directly to the dermatologist* based on the assessment with an app, without the need first to visit a GP. The possibility was thought to lead to an optimized flow of patients with high-risk lesions, thereby contributing to a streamlined patient journey.

*You will get very strange phenomena that patients might come walking in with the diagnosis. I won’t say that we should then become a garage company*,* but then we might start doing the excisions much more or the more complex things. That can also contribute to cost efficiency.“ – Derm*,* FG 17-12*.

### Preconditions

*Evidence-based verification of accuracy* was identified as the first precondition for endorsing mHealth apps for skin cancer triage among HCPs. While they agreed that apps should reach a high detection accuracy, there was no consensus in terms of specific accuracy standards that should be met. However, participants stressed that the accuracy of mHealth apps for skin cancer triage should be *tested by an independent organization* and not only by app developers themselves, as this was thought to improve the reliability of the evidence. Furthermore, dermatologists considered it essential t*o include a large representative sample in these validation studies*,* comparable to the intended user populations*. Dermatologists explained that including a different study population than the intended use population may result in the evidence not being representative and thus inaccurate.

*So the study population must also correspond to the population that will be using the product. That’s very important to me*,* it has to be large enough*,* of course*,* the study and it has to be independent people who have done that. And if this is done and then preferably at least two different groups*,* then I would have some confidence in that. Derm*,* FG 8-12*.

The second precondition perceived by HCPs was *successful integration within clinical practice*, consisting of four sub-preconditions. They perceived *appropriate communication of medical information* by the app as an essential element of successful integration within clinical practice. HCPs were fearful of the negative effects on patients if a diagnosis or severity would be communicated by an app. Instead, they suggested that a risk indication instead of diagnosis should be used, which could be presented in the form of an advice whether a doctor visit is necessary.

*More in the neutral terms I think*,* like: ‘consult this lesion with your doctor’*,* but not already making an assessment of what the severity is*,* because that makes some people*,* not everyone*,* but some people are very sensitive to that and I think we do need to take that into account. GP*,* FG 7-1*.

The second sub-precondition, which was specifically mentioned by dermatologists, was the possibility of sharing a report of a skin lesion from the app with the HCP. Dermatologists saw use in evaluating a skin triage report remotely, offering an intermediate step to consult a dermatologist after using an app to screen a lesion but before an in-person visit at the dermatologist. *Adequate protection of patient data* was identified as a third sub-precondition specifically for GPs. GPs emphasized that mHealth apps process medical data and should therefore ensure adequate protection of this data whilst maintaining a high privacy standard. The fourth and final sub-precondition supporting a successful integration in clinical practice identified for HCPs was *the involvement of HCPs during the implementation process*. Both types of HCPs stressed the importance of a well-thought-out implementation strategy and considered their involvement essential to ensure the proper positioning of an mHealth app in the healthcare system. A crucial element of this strategy was adequately informing colleagues through the national societies to ensure that all HCPs are aware of the functionalities, limitations, and risks of the triage apps that may be used by patients and the general population.

*Clarity about potential liability in case of adverse events* was identified as the third perceived precondition among HCPs for the endorsement of mHealth apps for skin cancer triage in the general population. HCPs were weary of the risks that may be associated with using an mHealth app and were concerned about liability issues. Therefore, clarity about potential liability was considered critical.

*If a patient uses the app independently*,* I think liability partly lies with the app. (…) But if it’s a professional tool and the doctor still sees the lesion*,* then I believe the doctor holds full responsibility. So if the patient uses it alone — that’s tricky. – GP FG 7-1*.

An accessible and inclusive app design was the fourth and final perceived precondition for endorsement of mHealth apps for skin cancer triage identified for GPs and consisted of five supporting sub-preconditions. To this end, apps should, first of all, include clear instructions, for example, on how to take a suitable photo with sufficient lighting. Furthermore, a quality check of skin lesion photos was considered a crucial element to guarantee that a proper assessment can be made by an app. Third, a simple user interface was thought to make apps easy to navigate. Fourth, GPs mentioned that an app should be tested by a diverse population in terms of age, skin type and physical disabilities.

*The corona tracing app is a good example. (…) All kinds of groups of people, including blind people, had to test that app first and the design was adapted. So that design part is very important, I think. - GP, FG 24-11*.

Finally, GPs mentioned that potential users should be informed properly about the potential impact on their privacy that may result from the use of an app by providing an *understandable data usage statement*.

## Discussion

This study explored HCPs’ views on mHealth skin cancer triage apps for the general population. We found that both dermatologists and GPs perceive considerable risks, including incorrect diagnoses and subpopulation exclusion, but also significant benefits, such as a streamlined patient journey for early cancer detection. In order for HCPs to actually endorse mHealth skin cancer apps, several preconditions which are currently unmet need to be addressed. Our study highlights the risk of incorrect diagnoses with mHealth apps in the general population, aligning with public concerns about accuracy [17, 18]. Recent validation studies report widely varying accuracy levels in skin cancer detection by these apps [8, 19, 20, 21]. Nevertheless, the adoption of skin cancer triage mHealth apps is rapidly increasing. While our study’s participating HCPs didn’t agree on a minimum required accuracy, they stressed the need for independent accuracy testing on a user-similar population. This raises a pressing question for medical device regulators in countries with these apps: Should their availability be restricted, and if so, what accuracy standards and testing conditions should be mandated? These concerns about diagnostic errors not only reflect limitations in current technologies, but also point to insufficient public awareness of skin cancer signs—highlighting the need for both stricter validation and improved education.

Recent literature highlights growing concerns about health disparities resulting from AI implementation in medicine [22–24]. In line with this, HCPs in our study identified the risk of excluding subpopulations based on digital literacy and skin color. Therefore, they emphasized the importance of an accessible and inclusive app design tested on a diverse population as a precondition for implementation to mitigate this risk. Despite the lower overall incidence of skin cancer among black individuals compared to white individuals, their diagnosis often occurs at later stages, with lower survival rates [25, 26]. This suggests that facilitating early detection could disproportionately benefit black patients individually, even though the overall impact may be limited due to their lower skin cancer incidence [27].

Balancing risks and benefits is pivotal in evaluating the usefulness of mHealth apps. Health systems globally face increasing skin cancer cases, underscoring the need for streamlined triage [28]. If AI smartphone apps attain dermatologist-level accuracy, they could enhance care access and detection precision, as emphasized by HCPs. Furthermore, the heightened public awareness and early detection mentioned in this study could potentially reduce incidence and improve skin cancer survival rates [29]. However, implementation remains delicate, with notable risks. To ensure success, measures like minimizing GP or other primary care providers’ autonomy and preserving diagnostic experience are necessary to avoid overshadowing the benefits.

The perceived preconditions for endorsement by HCPs may play a key role in guiding the mHealth implementation towards success. When comparing those to current practice, it appears that many preconditions are currently unmet. Adequate algorithm content, validation, and regulation are critically questioned by lacking validation and insufficient diagnostic accuracy, according to recent studies [8, 9]. Furthermore, national societies are not endorsing but actively warning against adverse events following the use of mHealth apps for skin cancer detection, and crucial questions remain in many countries about the liability for HCPs after an incorrect app advise [30, 31]. Addressing these perceived preconditions will be vital for successful implementation and warrants a tailored implementation strategy based on the views of key stakeholders, including the perspective of patients and the general population.

Beyond identifying perceived risks, benefits, and preconditions, the focus groups also yielded valuable insights that may inform future research and development of mHealth apps for skin cancer triage. Participants suggested that apps should include clear user instructions and automated quality checks for submitted images, to ensure usability and diagnostic reliability. Moreover, the importance of a simple and accessible interface, as well as inclusive testing across a diverse user population, was emphasized. These suggestions highlight key design features that warrant further investigation. Future studies could explore how such features influence user engagement, diagnostic accuracy, and equitable access to care, ideally in close collaboration with healthcare professionals to support meaningful clinical integration.

### Limitations and strengths

This study has limitations. Conducting online focus groups due to COVID-19 restrictions made it challenging to gauge participants’ body language, potentially missing signs of discomfort, disinterest, or agitation. However, this online approach facilitated the inclusion of a diverse group of HCPs from various Dutch regions, mitigating some of the limitations associated with remote data collection [32, 33]. Although we used purposive sampling and multiple recruitment channels—including social media, e-mail, and a professional society newsletter—to ensure diversity, we acknowledge that digital recruitment may have introduced some degree of selection bias toward digitally engaged professionals. Nevertheless, as shown in Supplementary eTable 1, the sample included a broad age range suggesting that the perspectives captured are not limited to those of younger individuals.

A strength of this study was its inclusion of dermatologists and GPs, representing primary, secondary, and tertiary care perspectives on mHealth skin cancer apps. Separate focus groups helped uncover potential differences in views, although there appeared to be substantial overlap in their perceptions of benefits, risks, and endorsement conditions. While recognizing that differences in healthcare systems should be taken into account, we believe that many of the themes identified in this study are transferable to settings outside the Netherlands [34]. Moreover, conducting this study in the Netherlands, where around 30% of adults have free access to skin cancer mHealth apps through their health insurer, likely provided insights based on real-world experiences, not just hypothetical scenarios. Nonetheless, as HCPs gain more experience and app functionalities evolve, revisiting their views in the future would be advisable to assess the sustainability of the implementation [35].

In summary, this study illuminates HCPs’ views on risks, benefits, and preconditions for the endorsement of mHealth apps integrated with artificial intelligence for skin cancer triage in the general population. These conditions appear to be largely unmet in current practice. Given HCPs’ crucial role and the app’s increasing availability, aligning design and implementation with their perspectives is imperative.

## Electronic supplementary material

Below is the link to the electronic supplementary material.

Supplementary Material 1

Supplementary Material 2

## Data Availability

The data that support the findings of this study are not publicly available due to privacy and ethical restrictions but may be available from the corresponding author (m.lugtenberg@erasmusmc.nl) on reasonable request.
