# Supplementary material for: Mobile health apps for skin cancer triage in the general population: a qualitative study on healthcare providers’ perspectives
Source: BMC Cancer. 2025 May 9;25:851. doi: 10.1186/s12885-025-14244-3 (PMC12065202; doi:10.1186/s12885-025-14244-3)
Supplement: Supplementary file 1 — Supplementary Material 1 [file 12885_2025_14244_MOESM1_ESM.docx]

| **Participant** | **Focus group** | **Age** | **Gender** | **Profession** | **Familiar with AI for skin cancer triage** | **Sees AI as an addition to skin cancer care** | **Would advise patients to use AI** |
| --- | --- | --- | --- | --- | --- | --- | --- |
| Participant 1 | 1 | 35 | Female | GP | No | Yes | Not yet, needs to be reliable |
| Participant 2 | 1 | 32 | Female | GP | No | Yes | Not yet |
| Participant 3 | 1 | 32 | Male | GP | - | Yes | Yes |
| Participant 4 | 1 | 32 | Male | GP | No | No | No |
| Participant 5 | 2 | 43 | Male | GP | No | Yes | Yes |
| Participant 6 | 2 | 42 | Male | GP | Yes | Yes | Wonders if he should advise it, or that it should be available elsewhere in the care process |
| Participant 7 | 2 | 36 | Male | GP | Yes | Yes | Yes |
| Participant 8 | 2 | 62 | Female | GP | No | Yes | Open to try a validated application |
| Participant 9 | 2 | 34 | Female | GP | Yes | Yes | Yes |
| Participant 10 | 3 | 51 | Male | Derm | Yes | Yes | Yes, but only selected applications |
| Participant 11 | 3 | 46 | Male | Derm | Yes | Yes | No, but is open to it |
| Participant 12 | 3 | 59 | Female | Derm | Yes | Yes | Informative, yes. Screening, no |
| Participant 13 | 3 | 41 | Female | Derm | Yes | Yes, as a tool for GP’s | Only a few applications |
| Participant 14 | 4 | 41 | Female | Derm | No | Yes | No |
| Participant 15 | 4 | - | Female | Derm | Yes | Yes | Not yet |
| Participant 16 | 4 | 35 | Male | Derm | Yes | - | No |
| Participant 17 | 4 | 52 | Male | Derm | Yes | Yes | Yes, provided that it is reliable |
| Participant 18 | 4 | - | Male | Derm | Yes | Yes | - |
| Participant 19 | 5 | 35 | Female | GP | No | Yes | No |
| Participant 20 | 5 | 31 | Male | GP | No | Yes | Yes |
| Participant 21 | 5 | 34 | Female | GP | No | Yes | Yes |
| Participant 22 | 5 | 32 | Male | GP | No | Yes | No |
| Participant 23 | 5 | 39 | Female | GP | Yes | Yes | Yes |
| Participant 24 | 5 | 32 | Female | GP | No | Yes, if research has proven effectiveness |  |
| Participant 25 | 5 | 35 | Female | GP | Yes | Yes | No |
| Participant 26 | 5 | 33 | Female | GP | Yes | Yes | Yes |
| Participant 27 | 6 | 40 | Male | Derm | Yes | Yes | Not for screening, just for specific lesions |
| Participant 28 | 6 | 48 | Female | Derm | Yes | Yes | Depends on quality |
| Participant 29 | 6 | 37 | Female | Derm | Yes | Yes, provided that it is reliable | No |
| Participant 30 | 6 | 33 | Male | Derm | Yes | Yes | For picture comparison over time. Not for diagnostics until sensitivity is 100% |
| Participant 31 | 6 | 41 | Female | Derm | Yes | Yes, provided that sensitivity and specificity are high | No |
| Participant 32 | 6 | 32 | Female | Derm | No | Yes | Yes |
| Participant 33 | 6 | - | Female | Derm | Yes | Yes | - |
|  |  |  |  |  |  |  |  |

**eTable 1.** Individual characteristics of focus group participants
