# Supplementary material for: Mobile health apps for skin cancer triage in the general population: a qualitative study on healthcare providers’ perspectives
Source: BMC Cancer. 2025 May 9;25:851. doi: 10.1186/s12885-025-14244-3 (PMC12065202; doi:10.1186/s12885-025-14244-3)
Supplement: Supplementary file 2 — Supplementary Material 2 [file 12885_2025_14244_MOESM2_ESM.docx]

**Supplement 1 - Focus group topic guide**

**Topic guide** Views of dermatologists and general practitioners towards AI in skin cancer care


**Introduction

Topic 1. Views towards the use of artificial intelligence in general healthcare**

- Knowledge of AI (applications) in healthcare (i.e., not specifically related to skin cancer)
- Perception of AI (applications) in healthcare
- Benefits and risks/barriers of AI (applications) in healthcare

**Topic 2. Views towards current skin cancer care practice**

- General views towards current skin cancer practice
- Perceived obstacles in the diagnosis, treatment and follow-up care of patients with skin cancer.

**Topic 3. Views towards the use of artificial intelligence by patients and the general population for skin cancer triage**

- Knowledge of AI (applications) in skin cancer triage
- Experiences with AI (applications) in skin cancer triage
- General views towards AI (applications) in skin cancer triage
- Reasons to start or refrain from using AI (applications) in skin cancer triage
- Requirements for using AI (applications) in skin cancer triage

**Concluding remarks**

**Supplement 2 – Questionnaire**

**Age:**

**Gender:**

**Profession:**

- - Dermatologist
  - General Practitioner

**Are you familiar with AI for skin cancer triage?**

- - Yes
  - No
  - Other:

**Do you see AI as an addition to skin cancer care?**

- - Yes
  - No
  - Other:

**Would you advise patients to use AI for skin cancer triage?**

- - Yes
  - No
  - Other:
